# Distally Tilted Implants According to the All-on-Four^®^ Treatment Concept for the Rehabilitation of Complete Edentulism: A 3.5-Year Retrospective Radiographic Study of Clinical Outcomes and Marginal Bone Level Changes

**DOI:** 10.3390/dj10050082

**Published:** 2022-05-11

**Authors:** Árpád László Szabó, Ádám László Nagy, Csaba Lászlófy, Márió Gajdács, Péter Bencsik, Krisztina Kárpáti, Zoltán Baráth

**Affiliations:** 1 Department of Prosthodontics, Faculty of Dentistry, University of Szeged, Tisza Lajos körút 62-64, 6720 Szeged, Hungary; szabo.arpad@stoma.szote.u-szeged.hu (Á.L.S.); nagy.adam.laszlo@stoma.szote.u-szeged.hu (Á.L.N.); 2 Oral Centrum Dental Ltd., Angyalka u. 1/A, 2030 Érd, Hungary; 3 Sanoral Healthcare Ltd., Ó utca 6, 1066 Budapest, Hungary; private@drlaszlofycsaba.hu; 4 Department of Oral Biology and Experimental Dental Research, Faculty of Dentistry, University of Szeged, Tisza Lajos krt. 63., 6720 Szeged, Hungary; mariopharma92@gmail.com; 5 Department of Pharmacology and Pharmacotherapy, Albert Szent-Györgyi Medical School, University of Szeged, Dóm tér 12, 6721 Szeged, Hungary; bencsik.peter@med.u-szeged.hu; 6 Department of Orthodontics and Pediatric Dentistry, Faculty of Dentistry, University of Szeged, Tisza Lajos körút 62-64, 6720 Szeged, Hungary; drkarpatik@gmail.com

**Keywords:** immediate implant placement, dental implants, alveolar bone resorption, All-on-Four™, cone-beam computed tomography, clinical study

## Abstract

Bone grafting procedures during the use of dental implants may be avoided by the use of tilted implants in the maxilla and the mandible; advantages of angled implants are associated with the extension of the distal cantilever, leading to better implant survival rates. However, the bending effect on the single tilting implants may increase the marginal bone stress. The purpose of the present study was to retrospectively assess the clinical success and proximal bone loss rate following the implantation of distally tilted implants according to the All-on-Four™ prosthetic concept—based on radiographic findings—in a single-center experience, in addition to the assessment of the outcomes in the context of various clinico-epidemiological correlates. During the study period, *n* = 36 patients (24 males and 12 females) with complete records of periapical radiographs, received a full-arch fixed bridge supported by two axial and two distal tilted implants; overall *n* = 144 and *n* = 144 implants (Nobel B) were place in the maxilla and mandibles of patients, respectively. Mean age of patients at the time of fixture installation was 58.75 ± 13.71 years; *n* =11 patients presented with relevant underlying conditions/habits. To assess peri-implant bone-level changes, matched and calibrated orthopantomogram (OPT) images were taken at follow-ups after 1.5 years, 2.5 years, and 3.5 years post-restoration, and marginal bone levels were assessed on the mesio- (MA) and disto-approximal (DA) aspects. All implants were successful, resulting in a 100% overall survival rate. The radiographic mean bone loss levels at baseline (mean ± SEM) were 0.181 ± 0.011 mm and 0.178 ± 0.017 mm in the maxilla and mandible, respectively, while by the 3.5-year mark, bone loss was 0.770 ± 0.029 mm and 0.713 ± 0.026 mm in the maxilla and mandible (*p* > 0.05), respectively; bone-level changes were significant over time (*p* = 0.035 and *p* = 0.033). Peri-implant bone loss was more aggressive around titled distal implants versus mesial actual position implants. The effect of smoking and other underlying conditions showed significantly higher (*p* < 0.05) bone resorption levels when assessed on an individual implant-level, while during patient-level analysis, only a tendency was shown for higher bone loss rates for both MA and DA implants (*p* > 0.05). Within its limitations, our study has concluded that the use of All-on-Four™ prosthetic concept for total arch rehabilitation yields higher bone loss in association with tilted implants and, in some cases, on the MA surfaces at vertically positioned implants after >40 months in function.

## 1. Introduction

Disorders of the oral cavity are a significant cause of decreased quality of life (QoL) and they are important contributors to years lived with disability (YLDs), affecting facial aesthetics, and the capacity to eat or speak [1]. Partial or complete edentulism (the latter exceeding >10% in patients aged 50 years or older globally) is a definite condition—occurring as a long-term consequence of dental caries and periodontal disease—which has emerged as a global public health issue [2,3]. People with low socio-economic standing are disproportionally affected by tooth loss; in addition to bad oral hygiene, many modifiable risk factors (e.g., diets rich in carbohydrates, tobacco consumption, and alcohol use)—relevant in the development of other non-communicable diseases—are also critical in leading to edentulism [4,5]. The oral rehabilitation of edentulous patients involves the use of dental implants. Rehabilitation of total edentulism with a conventional implant-supported fixed restoration involves bone grafting, a sinus elevation procedure, and soft tissue management, in case of a severely atrophic alveolar process [6]. However, bone augmentation carries considerable risks of complications (infection, loss of soft tissue contours and/or volume, graft failure, and risks associated with donor materials), procedural issues, and patient morbidity, and the reconstructive surgery corresponds to higher costs and longer recovery time intervals [7,8].

This anatomical limitation may be treated with a long distal cantilever, short implants, or implants placed in a specific anatomical area [9]. Avoiding bone grafting procedures by using tilted implants in the maxilla and the mandible is a recognized alternative with no significant clinical difference in success rates compared to axially placed implants, and their acceptability by patients is also higher [10]. Clinical advantages of angled implants are associated with the extension of the distal cantilever, leading to better implant survival rates [11]. The bending effect on the single tilting implants may increase the marginal bone stress, but this may be augmented with splinting them into a multiple implant-supported prosthesis, according to a two-dimensional finite element analysis [12]. It has been shown that cervical bone stress increases proportionally with the length of the cantilever, while it is not influenced by the length of the implants. Marginal bone loss between tilted and axially placed implants demonstrated no difference and presented with no detrimental effects on osseointegration levels [13].

The principle of the All-on-Four™ concept is to apply four implants in the anterior part of completely edentulous jaws to support a provisional, fixed, and immediately loaded prosthesis [14]. During implantation on the All-on-Four™ methodology, the rehabilitation of the total arch is carried out without the need for bone augmentation and fixed prosthesis supported by two axial implants in the anterior segment, and one tilted implant anteriorly to the mental foramina and the anterior lobe of sinus on each posterior segment [15]. The tilted implants provide anchorage for the first molar occlusion with a short cantilevered segment, by reaching a more posterior implant position [16]. Maxillary and mandibular All-on-Four™ rehabilitations have a comparable cumulative survival rate [17]. Based on biomechanical analyses, major cantilever loading is the highest at the most anterior and posterior implants supporting a reconstruction, irrespective of the intermediate implants. The load supported by the most heavily loaded implant in fix restoration is independent of the number of complementary implants, according to in vivo measurements [18].

The purpose of the present study was to retrospectively assess the clinical success and the rate of proximal bone loss following the implantation of distally tilted implants according to the All-on-Four™ prosthetic concept—based on radiographic findings—in a single-center experience, in addition to the assessment of the outcomes in the context of various clinico-epidemiological correlates (e.g., age, oral hygiene, parafunctional habits, and smoking habits of the patients). Our initial hypotheses were: (i) no differences in peri-implant bone levels among axial and tilted implants during follow-ups; and (ii) no differences in peri-implant bone levels measured at the mesio-approximal and disto-approximal aspects of implants during follow-ups.

## 2. Materials and Methods

### 2.1. Study Design

The present single-center retrospective study aimed to evaluate the clinico-epidemiological and radiographic data (peri-implant bone-level changes) longitudinally from patients undergoing an implant surgical procedure with an immediately-loaded, four-implant-supported fixed prosthetic concept, following the All-on-Four™ protocol, between 1 January 2017 and 1 January 2022.

### 2.2. Inclusion Criteria

The study employed convenience sampling at the study center. The following inclusion criteria were set for the study:

(i). Patients aged 18 years or older;
(ii). Patients in an overall good health condition, able to undergo surgical intervention;
(iii). Patients in need for a complete rehabilitation of the edentulous maxilla or mandible, and the possibility of placing a minimum of 4 implants (at least 10 mm long);
(iv). Sufficient bone height in the sites intended for the placement of implants (min. 6 mm, evaluated by preoperative CT scans analysis).

The exclusion criteria for the study were:

(i). Presence of an acute infection at the planned implant sites;
(ii). Known coagulopathies or other hematologic diseases;
(iii). Recent occurrence of severe cardiovascular or cerebrovascular event;
(iv). Diseases affecting the immune system;
(v). Uncontrolled diabetes mellitus (DM);
(vi). Pregnancy or lactation;
(vii). Metabolic illnesses affecting the bones, bisphosphonate therapy;
(viii). Heavy smoking (>10 packs/day);
(ix). Systemic chemotherapy or irradiation of the head and neck region within the last 12 months;
(x). Presence of parafunctional habits, such as severe bruxism or clenching (assessed and identified by the clinicians, based on clinical signs and symptoms);
(xi). Inadequate oral hygiene level (full-mouth plaque and bleeding scores over 20%), poor perceived motivation on the part of the patient to maintain good oral hygiene throughout the study.

### 2.3. Preoperative Treatment

Prior to surgical treatment, the patients’ medical and dental histories, relevant lifestyle habits (e.g., smoking), and potential drug allergies were reviewed; the preoperative assessment of the patients was carried out by a prosthodontist and a periodontist. Following the presentation of the treatment plan to the patients and obtaining consent, surgical treatment was scheduled. Cone-beam computed tomography (CBCT) scans (i-CAT cone beam CT-scanner, Imaging Science) were carried out for preoperative assessment. Individuals followed an antibiotic regimen per os (clindamycin 300 mg q.i.d.) three days prior to the surgical procedures in cases where teeth had to be extracted simultaneously. Preceding surgery, local anesthesia was administered (4% articaine containing 1:100,000 epinephrine).

### 2.4. Implant Placement Protocol

All relevant operative interventions were performed by the same surgeon with more than twenty years of experience associated with immediate loading procedures. Quantitative and qualitative assessment of the jaw bone was performed by means of preoperative radiographs, visual inspection, and tactile evaluation during drilling; appraisal of bone quality was carried out using CBCT scans. Each individual received (i) 2 distally tilted implants in the posterior region and, after that, (ii) 2 anterior implants in the maxilla or the mandible. In the maxilla, tilted implants were positioned just anterior to the maxillary sinus, while in the mandible they were positioned anterior to the mental foramen. The placement of implants was according to the All-on-Four™ treatment concept, using the All-on-Four™ surgical guide (Nobel Biocare; Kloten, Switzerland); comprehensive details regarding the procedure have been described elsewhere [19]. Regarding bone regeneration, universal clinical protocols for immediate implant placement were used [20]. Localized bone grafting was performed to cover exposed threads and/or other osseous defects associated with extraction sockets, as needed with demineralized allografts. For the fabrication of the master cast to create the patients’ provisional restoration, open-tray multi-unit impression copings were placed on the multi-unit abutments to make an impression using precision impression material (Flexitime, Heraeus Kulzer, Hanau, Germany).

Following the operative procedure, patients were instructed to abstain from brushing in the first 7 days post-op, and to rinse using warm water. For 24 h post-io, instructions and recommendations were given for a soft diet (cold or at room temperature), to be followed by a semisolid diet for the following three months. Patients were supplied with antibiotics (amoxicillin 500 mg t.i.d. or clindamycin 300 mg t.i.d. for seven days) and analgesics (non-steroid anti-inflammatory drugs) to control post-operative pain and inflammation as per standard guidelines and protocols in oral surgery. To confirm implant positions, and the positions of the prosthetic components, a CBCT scan was taken immediately postoperatively.

### 2.5. Restorative Protocol

Prior to the surgical intervention, a heat-cured acrylic resin (Ivocap High Impact acrylic, Ivoclar Vivadent, Amherst, NY, USA) was prefabricated, which was amended to the master model directly after the surgery. Fabrication was carried out using cold curing material (Probase, Ivoclar Vivadent, Amherst, NY, USA). Following 3–4 h after the completion of the operation, the provisional all-acrylic prosthesis was seated. Routine follow-ups were scheduled for the patients after surgery at 7, 14, and 28 days and 3 months after surgery, and on a yearly basis thereafter. Following the 3-month appointment, fabrication of the definitive prosthesis was initiated, consisting of a milled titanium frame with a wrap-around heat-cured acrylic resin (Nobel Procera Implant Bridge titanium framework veneered with composite). The antagonist denture was a fixed denture/implant supported restoration in all cases. A long-cone paralleling method was applied to obtain matched and calibrated orthopantomogram (OPT; panoramic X-ray) images at the 3-month appointment and at the subsequent appointments continuously. The 3-month radiographs after the time of placement of the definitive prosthesis were utilized as a baseline (T_0_) to assess the bone levels longitudinally. At the respective follow-ups, the implants were assessed for signs of peri-implantitis, plaque, and bleeding on probing (BOP), based on clinical routine guidelines.

### 2.6. Radiographic Assessment: Calculation of Marginal Bone Loss

Peri-implant bone-level changes were measured by matched and calibrated OPT images taken at the 3-month appointment (i.e., baseline) and follow-ups after 18 months (T_1_; 1.5 years post-restoration), 30 months (T_2_; 2.5 years post-restoration), and 42 months (T_3_; 3.5 years post-restoration); marginal bone level (the most coronal bone-to-implant contact) was assessed on the mesio- (MA) and disto-approximal (DA) aspects. An independent researcher—not affiliated with the primary center and investigators—evaluated the OPT images. Radiographs were digitized in a 640 (H) × 480 (V) pixel matrix image with an 8-bit depth. The density and contrast were then adjusted for optimal visualization of the marginal bone, and the digital images were saved as a TIF extension image. The 2D images were then exported and analyzed using the CLINIVIEW image analysis software (MI Dental, Knowsley, Prescot, UK). Calibration for image analysis was performed on an individual implant-level (*n* = 288) to achieve the most accurate results possible, where the known size and specifications of the individual documented implants were used as the basis for calibration to allow for the calculation of marginal bone level changes in the area. Assessment of bone levels were carried out and captured separately on the MA and DA sides of the implant. The change in marginal bone levels (ΔBL (mm)) from the baseline (T_0_) to the values recorded at the follow-ups T_1_, T_2_, and T_3_ were calculated.

### 2.7. Outcome Variables Assessed

The following outcome variables were ascertained during the study:

(a). Survival of implants (%): defined as implants being stable and functional (implant stability was assessed using pressure from two opposing instruments following the unscrewing of the prosthesis), lack of peri-implant radiolucency on radiographs, lack of suppuration or pain associated with the implant site, no signs of peri-implantitis, and lack of neuropathies or persistent paresthesia.
(b). Changes in marginal bone levels (ΔBL (mm)) from the baseline (T_0_) to the values recorded at the follow-ups T_1_, T_2_, and T_3_ post-implantation.

Marginal bone level changes were studied in the context of the following correlates and sub-groups:

(i). Maxillary vs. mandibular implants;
(ii). Tilted (posterior) and axial (anterior) implants;
(iii). Mesio- (MA) and disto-approximal (DA) aspects of implants;
(iv). Patients presenting with underlying conditions/parafunctional habits.

### 2.8. Statistical Analysis

Descriptive statistics (including means ± SEM (standard error of the mean), ranges and percentages) was performed using Microsoft Excel 365 (Microsoft Corp., Redmond, WA, USA). Statistical analyses were carried out by the SPSS v. 22.0 (IBM Corp., Endicott, NY, USA): the normality of variables was tested using the Shapiro–Wilk test; inferential statistics were performed using independent-sample *t*-test, one-way ANOVA with Tukey’s post hoc test and Pearson’s correlation. *p* values < 0.05 were considered statistically significant.

### 2.9. Ethical Considerations

The study was conducted in accordance with the Declaration of Helsinki and national and institutional ethical standards. Ethical approval for the study protocol was obtained from the Human Institutional and Regional Biomedical Research Ethics Committee, University of Szeged (registration number: 158/2021-SZTE [5035]). All participants were informed of the nature and aims of the study and the data collected; all participants of the study signed an informed consent form.

## 3. Results

### 3.1. Patient Characteristics, Clinical Outcome

During the study period, *n* = 36 patients (24 males and 12 females) with complete records of periapical radiographs underwent implant placement using the All-on-Four^™^ concept and have been rehabilitated; overall *n* = 144 and *n* = 144 implants (Nobel B) were place in the maxilla and mandibles of patients, respectively, i.e., the analysis of individual implant data for *n* = 288 was performed. The mean age of patients at the time of fixture installation was 58.75 ± 13.71 years (range: 19–90 years). Out of the thirty-six patients involved, six patients receiving implants in the mandible (controlled DM *n* = 1, mild bruxism *n* = 1, impacted oral hygiene (i.e., full-mouth plaque score and full mouth bleeding score 0–20%) *n* = 1, smoking *n* = 3, smoking and impacted oral hygiene *n* = 1) and five patients receiving implants in the maxilla (controlled DM *n* = 1, impacted oral hygiene *n* = 1, mild bruxism *n* = 1 and smoking *n* = 1, smoking and impacted oral hygiene *n* = 1) had underlying conditions/habits relevant to the outcome of the study (these patients will be grouped together for subgroup analyses). Among smokers, the daily average tobacco consumption of was 8.2 ± 2.6 cigarettes. During the 42-month study period no implants have failed, resulting in 100% overall survival rate (not affected by the clinico-epidemiological parameters of the patients), highlighting the success of the All-on-Four^™^ concept. All patients complied with the set timetables, no patients (*n* = 0) were lost to follow-up at either follow-ups (at 18 months, 30 months, and 42 months post-restoration); the status of all *n* = 36 patients were followed for the entirety of the study period.

### 3.2. Marginal Bone-Level Changes across Different Correlates

The radiographic mean bone loss levels at baseline (T_0_) were 0.181 ± 0.011 mm (mean ± SEM; maxilla (*n* = 144): 0.178 ± 0.017 mm vs. mandible (*n* = 144): 0.184 ± 0.015 mm; *p* > 0.05); in the subsequent analyses, marginal bone level changes (ΔBL) at T_1_, T_2_, and T_3_ follow-up times were compared to these initial values. Levels of marginal bone loss according to different correlates are presented in Table 1 (maxilla vs. mandible), Table 2 (axial vs. posterior implants) and Table 3 (MA vs. DA); in addition, the extent of bone loss on an individual implant-level is represented in Table 4 and Table 5.

The average rate of bone loss after the 1.5-year follow-up was 0.558 ± 0.029 mm and 0.484 ± 0.024 mm, while by the 3.5-year mark, bone loss was 0.770 ± 0.029 mm and 0.713 ± 0.026 mm regarding the implants placed in the maxilla and mandibular bone, respectively; bone-level changes were significant over time (*p* = 0.035 and *p* = 0.033, respectively), while the alterations observed around the maxilla and mandibular implants did not differ significantly (*p* > 0.05) (Table 1). In patients presenting with underlying conditions/habits (described previously), a tendency was shown for higher bone loss rates in the maxilla (T1: −0.633 ± 0.056 mm, T2: −0.780 ± 0.056 mm, and T3: −0.830 ± 0.053 mm) and the mandible (T1: −0.535 ± 0.048 mm, T2: −0.700 ± 0.054 mm, and T3: −0.763 ± 0.051 mm), however none of these differences were statistically significant (*p* > 0.05).

Measured bone loss was significantly higher in posterior implants throughout the follow-up period (Table 2); in addition, bone-level changes were significant over time (*p* = 0.041 and *p* = 0.039). Similarly to the previous result, in patients presenting with underlying conditions/habits, bone loss rates that are numerically higher—but not statistically significant—were observed for axial (T1: −0.422 ± 0.038 mm, T2: −0.596 ± 0.038 mm, and T3: −0.641 ± 0.036 mm) implants, while bone loss was significantly higher for tilted (T1: −0.733 ± 0.056 mm, T2: −0.907 ± 0.061 mm, and T3: −0.949 ± 0.057 mm) implants (*p* < 0.05).

No significant differences were observed in the measured bone-level changes on the MA and DA aspects of the implants throughout the study period (*p* > 0.05 in all cases; Table 3), while bone loss increased consistently during the follow-up periods in both the MA (*p* = 0.029) and DA (*p* = 0.035) aspects. During subgroup analysis, a tendency was shown for higher bone loss rates for both MA (T1: −0.586 ± 0.043, T2: −0.716 ± 0.046, and T3: −0.767 ± 0.042) and DA (T1: −0.545 ± 0.051, T2: −0.757 ± 0.063, and T3: −0.825 ± 0.060), however these differences were not statistically significant (*p* > 0.05).

The degree of bone resorption was also assessed on an individual implant-level, separately in the maxilla and mandible, presented in Table 4 and Table 5, respectively. In the case of the maxilla, higher bone loss was observed for the teeth 14DA (with −1.001 ± 0.101 mm at T3, range: −0.3–1.7 mm) and 24DA (with −1.066 ± 0.081 mm at T3, range: −0.6–1.8 mm), which were significantly higher than the values compared to most other teeth (*p* < 0.05) (Table 4). The highest rates of bone loss in the mandibles were shown for the teeth 34DA (with −0.872 ± 0.044 mm at T3, range: −0.6–1.3 mm) and 44MA (with −0.789 ± 0.066 mm at T3, range: −0.4–1.5 mm); bone resorption rate was significantly higher at 34DA than that observed for other teeth (*p* < 0.05), with the exception of 32MA and 44MA. Significantly increasing levels of bone loss were seen in all respective cases, both for maxilla and mandibular implants (*p* < 0.05). The effects of smoking and other underlying conditions were also assessed on these results; although we had a limited number of patients to pool from for aggregated data, significantly higher (*p* < 0.05) bone resorption levels were observed for 14MA (T1: −0.760 ± 0.137, T2: −0.900 ± 0.129, and T3: −0.940 ± 0.117), 24DA (T1: −1.100 ± 0.231, T2: −1.240 ± 0.211, and T3: −1.260 ± 0.219) and 44DA (T1: −0.700 ± 0.143, T2: −1.050 ± 0.183, and T3: −1.117 ± 0.168), while only numerical tendencies were shown for the other teeth.

We have tested whether the age of the patients was a relevant correlate regarding bone resorption levels; overall, we did not find any significant linear correlation between the degree of bone resorption and age. However, in case of 12MA in the maxilla, a positive (but non-significant) tendency could be observed.

## 4. Discussion

Following the introduction of dental implants, their stability may be characterized by two distinct processes: primary (mechanical) implant stability influences the immediate outcome of the surgery (influenced by bone quality, preparation of the implant bed, and implant geometry), while secondary (biological) stability is a dynamic physiological process (influenced by the underlying factors of the patients and implant microtopography), leading to the formation of the implant-bone interface [21]. Previously, conventional loading protocols were carries out after a healing period of 2–3 months; however, recently, the immediate loading protocols (highly relying on implants reaching high primary stability) have been extensively investigated for their clinical applicability, showing that their overall survival rates and patient satisfaction are comparable to two-stage protocols [22]. Only around two-thirds of patients are completely complication-free following the restoration of the implant-supported fixed prostheses; these complications may include biological adverse events (e.g., peri-implantits or loss of alveolar bone) and technical complications (screw loosening, retention loss, or fractures in the superstructures), that may lead to implant failure [7,23,24]. The clinical utility of the All-on-Four™ treatment concept has been demonstrated in numerous clinical studies, showing that this technique is distinguished by a predictable, positive prognosis and high patient satisfaction rates [14,25]. The superiority of this concept is associated with the implementation of an atrophic maxilla or mandible, less complicated surgery and upkeep, and masticatory forces in the satisfactory range [14].

The principal aim of the present retrospective study was to provide data on the clinical outcomes associated with distally tilted implants according to the All-on-Four™ therapeutic concept, and to assess the rates of marginal bone loss as a function of time elapsed and patient characteristics using radiographic findings. Various procedures preceding implant placement (e.g., impression, drilling, and introduction of tools) lead to inflammation and, consequently, a baseline level of bone resorption will inevitably occur [26]. Additionally, current publications provide evidence that repeated abutment manipulation—in case of implants with platform-switching—leads to detrimental changes in soft and hard tissues (i.e., tissue remodeling as measured by mucosal margins, implant shoulder, apical extension of the long junctional epithelium and most coronal bone-level in contact with the implant) [27]. Thus, on one hand, the use of implants with fixed abutments (“one abutment–one time” concept) may greatly stabilize peri-implant soft and hard tissues, while immediate implant placement may significantly reduce the initial unavoidable bone loss [28].

The 3.5-year-long follow-up period involved thirty-six patients, with an overall survival rate of 100%. High implant survival rates have been consistently reported for this technique; a comprehensive clinical study by Maló et al. reported a 98.2% cumulative implant survival rate, while based on the literature summary performed by Durkan et al., the overall success rate ranges between 92.2–100%, with no differences between tilted and axial implants in clinical success rates [14,29]. Based on our measured bone loss levels at baseline (T_0_; ~0.18 mm) and at the three follow-up points (T_1_, T_2_, and T_3_), bone loss showed the kinetics characteristic for a saturation curve, i.e., showing relatively high ΔBL values at the first-follow-up, with bone-levels changes “flattening out” the curve. By the third follow-up, mean bone loss in our patients was around 0.7–0.8 mm in both the maxilla and mandible, with specific positions in the maxilla and the mandible disproportionally affected; while a tendency for higher measured peri-implant bone loss was seen on the DA aspects, no significant differences were shown MA vs. DA aspects during follow-ups. Bone resorption measured on the MA aspects may be mediated by the chewing forces generated on extension surfaces and the negative torque generated by the bucco-lingual forces, which exerts tensile stresses on these surfaces (previously verified via finite element analyses), which can enhance bone resorption [30]. The cortical bone is most mechanically resistant to compressive forces, less resistant to tensile force, and the least resistant to shear forces [31].

The literature shows wide variation for bone-loss among studies, which is also considerably influenced by the follow-up period associated with the study. Similar kinetics in bone-loss where observed by Hürzeler et al. [32], showing marginal bone loss levels of 1.5 mm in the first year post-implantation, followed by 0.2 ± 0.5 mm in the subsequent years (in a 5-year follow up study), and Widmark et al. [33], with bone loss levels of 1.0 mm in the first year post-implantation, followed by 0.2 mm in the subsequent years (with follow-ups ranging between 3–5 years). In a study involving thirty-nine patients, Makary et al. assessed the clinical success of an early loading protocol by controlling for thread depth according to the bone density of the implant site. They showed a decrease in Implant Stability Quotient (ISQ) values in the early periods of healing—associated with the transition from mechanical to biological stability—following the surgical intervention, with the average marginal bone loss recorded at 0.12 ± 0.12 mm at 12 months post-loading with a 100% survival rate. No differences were shown between the MA and DA aspects of implants [34]. Similarly to this study, no differences were observed between bone loss at the MA and DA aspects by Barone et al. (0.4 mm vs. 0.5 mm) and Iasella et al. (1.0 mm vs. 0.8 mm) [35]. In a retrospective, CBCT-based study, Roe et al. reported a 0.82 ± 0.64 mm vertical, and 1.23 ± 0.75 mm horizontal bone height reduction at 1-year follow-up after immediate implant placement [36]. Interestingly, the study of Maló et al. reported implant failure in similar positions where our study presented with the highest levels on an individual implant-level [37].

On the other hand, bone loss levels were significantly higher around tilted implants compared to axial implants at every time-point. Tilted or short implants provide viable alternatives to bone grafting; on the other hand, they may lead to increased stress on the surrounding bone due to bending [12,38]. A finite-element analysis performed by Almeida et al. showed that the presence of distally tilted implants in an All-on-Four™ concept would result in higher maxillary bone stress compared to vertical implants [21]; these results were corroborated by the study of Rubo et al., additionally highlighting that the proportion of increased stress in proportional to the increased length of the cantilever [39]. In contrast, the paper by Durkan et al. reports bone loss levels within the range of 0.34–1.14 mm for axial, and 0.43–1.13 mm for angled implants, with no significant differences between them [14]. Implant length may also considerably affect implant survival and marginal bone loss, as demonstrated by the meta-analysis conducted by Fernandes et al.: based on the randomized controlled studies (RCTs) included in the analysis, survival rate of extra short (≤6 mm) and longer (6 mm) implants were similar (93.12% vs. 95.98%). In addition, average marginal bone loss at 1-year-, 3-year-, 5-year- and 8-year follow-ups were −0.71 mm, −0.42 mm, −0.69 mm, and −1.58 mm for extra short implants, while −0.92 mm, −0.43 mm, −0.46 mm, and −2.46 mm for longer implants, respectively. Overall, published clinical studies have shown that bone loss was lower in extra short implants [40]

As an additional aim of this research, the bone loss levels in patients presenting with underlying conditions and lifestyle factors were assessed as a sub-group within the patients involved in this study. It is well-known that inadequate oral hygiene (and a lack of motivation to practice good oral care), chronic conditions affecting the physiology of the oral cavity, smoking, and bruxism have a considerable influence on implant survival and clinical outcome, so much so that severe cases of the above mentioned are considered contraindications for the use of dental implants [41]. Among the patients, eleven were impacted by of these factors, which were also analyzed separately: while a tendency for higher bone loss values were shown around the implants in these individuals, significant differences were not shown. A retrospective, comparative study by Chrcanovic et al. found that the odds of implant failure was almost three times higher (OR: 2.71; 95% CI: 1.25, 5.88) in bruxers compared to non-bruxers; however, the level of differences showed a decreasing trend with increases in implant length and in cases of rough-surface implants [42]. The study of Glasuer et al. came to similar conclusions, with implant failure being 4.9-times more common (95% CI: 1.75, 13.71) in identified bruxers [43].

The study of Mohanty et al. classified patients based on the presence of the potential underlying factors affecting implant health: they have found that out of *n* = 425 dental implants studied, implant failure was observed in 29%, 27%, 15.2%, and 13% in the diabetes group, smoking group, periodontitis group, and the bruxism group, respectively [44]. Marginal bone loss levels around implants placed in maxillary sinus grafts were studied by Herzberg et al. in 4.5-year follow-up: their results showed that immediate loading was associated with lower baseline bone loss (0.08 ± 0.24 mm vs. delayed implantation 0.31 ± 0.62 mm), smoking considerably affected the average yearly marginal bone loss (0.24 ± 0.49 mm vs. non-smokers, 0.09 ± 0.32 mm). This study—showing a 95.5% cumulative survival rate—also highlighted the significant association between smoking status and >0.2 mm/year bone loss (*p* = 0.011), while the number of cigarettes did not have a modifying effect; no such association was seen for bruxism [45]. In a 5-year retrospective cohort study involving 100 smoking and 100 non-smoking patients, Maló et al. assessed the outcome of full-arch mandibular fixed prosthetic rehabilitations based on the All-on-Four™ concept: implant survival did not differ among the two groups (96.9% vs. 99.0%), while marginal bone resorption rate was significantly higher among smokers (1.68 ± 0.76 mm vs. non-smokers 1.98 ± 1.02 mm; *p* = 0.045); on the other hand, relevant differences in bone resorption around and anterior and posterior implant were not shown [46].

## 5. Conclusions

Within its limitations (i.e., retrospective study design, number of subjects, and follow-up period), our study has concluded that the use of All-on-Four™ prosthetic concept for total arch rehabilitation yields higher bone loss in association with tilted implants and, in some cases, on the MA surfaces at vertically positioned implants after >40 months of function. In addition, we have shown that inadequate oral hygiene, chronic conditions affecting the physiology of the oral cavity, smoking, and bruxism have a considerable aggravating role in enhancing marginal bone loss over time, increasing the risk for complications and implant failure. Overall, our present study highlights the areas of concern during prosthetic rehabilitation with the All-on-Four™ concept; in the future, results of our study may be extended as a randomized, controlled trial (RCT) with a longer follow-up period and larger number of patients.

## Figures and Tables

**Table 1:** Marginal bone-level changes around implants located in the maxilla and mandible during the 42-month study period.

	Marginal Bone Level Changes (ΔBL) (mm ± SEM)		
Follow-Up	Maxilla (*n* = 144)	Mandible (*n* = 144)	*p*-value (between groups) **
T1	−0.558 ± 0.029 ^a^	−0.484 ± 0.024 ^a^	*p* > 0.05
T2	−0.747 ± 0.030 ^b^	−0.678 ± 0.036 ^b^	*p* > 0.05
T3	−0.770 ± 0.029 ^b^	−0.713 ± 0.026 ^b^	*p* > 0.05
*p*-value (between follow-ups) *	***p = 0.035***	***p = 0.033***	

* based on ANOVA analysis, significant differences (*p* < 0.05) among groups (as demonstrated by post hoc tests) are indicated by different superscript letters (a and b); ** based on independent-sample *t*-test; *p*-values below 0.05 are shown in ***boldface*.**

**Table 2:** Marginal bone-level changes around axial and tilted implants during the 42-month study period.

	Marginal Bone Level Changes (ΔBL) (mm ± SEM)		
Follow-Up	Axial (Anterior) (*n* = 144)	Tilted (Posterior) (*n* = 144)	*p*-value (between groups) **
T1	−0.405 ± 0.021 ^a^	−0.637 ± 0.027 ^a^	***p = 0.008***
T2	−0.592 ± 0.024 ^b^	−0.676 ± 0.028 ^a^	***p = 0.048***
T3	−0.606 ± 0.022 ^b^	−0.833 ± 0.029 ^b^	***p = 0.002***
*p*-value (between follow-ups) *	***p = 0.041***	***p = 0.039***	

* based on ANOVA analysis, significant differences (*p* < 0.05) among groups (as demonstrated by post hoc tests) are indicated by different superscript letters (a and b); ** based on independent-sample *t*-test; *p*-values below 0.05 are shown in ***boldface*.**

**Table 3:** Marginal bone-level changes on the mesio- (MA) and disto-approximal (DA) aspects of the implants during the 42-month study period.

	Marginal Bone Level Changes (ΔBL) (mm ± SEM)		
Follow-Up	Mesio-Approximal (MA) Aspect (*n* = 144)	Disto-Approximal (DA) Aspect (*n* = 144)	*p*-value (between groups) **
T1	−0.519 ± 0.024 ^a^	−0.522 ± 0.029 ^a^	*p* > 0.05
T2	−0.697 ± 0.025 ^b^	−0.728 ± 0.032 ^b^	*p* > 0.05
T3	−0.729 ± 0.024 ^b^	−0.793 ± 0.029 ^b^	*p* > 0.05
*p*-value (between follow-ups) *	***p = 0.029***	***p = 0.035***	

* based on ANOVA analysis, significant differences (*p* < 0.05) among groups (as demonstrated by post hoc tests) are indicated by different superscript letters (a and b); ** based on independent-sample *t*-test; *p*-values below 0.05 are shown in ***boldface*.**

**Table 4:** Marginal bone-level changes around individual implants in the maxilla during the 42-month study period.

	Marginal Bone Level Changes (ΔBL) (mm ± SEM)							
Follow-Up	12DA (*n* = 18)	12MA (*n* = 18)	14DA (*n* = 18)	14MA (*n* = 18)	22DA (*n* = 18)	22MA (*n* = 18)	24DA (*n* = 18)	24MA (*n* = 18)
T1	−0.378 ± 0.051 ^a^	−0.489 ± 0.063 ^a^	−0.728 ± 0.093 ^a^	−0.567 ± 0.074 ^a^	−0.361 ± 0.061 ^a^	−0.439 ± 0.055 ^a^	−0.844 ± 0.095 ^a^	−0.538 ± 0.053 ^a^
Range (mm)	−0.0–0.7	−0.0–1.1	−0.2–1.4	−0.0–1.2	−0.0–0.8	−0.0–1.0	−0.4–1.8	−0.0–1.2
T2	−0.583 ± 0.042 ^b^	−0.661 ± 0.051 ^b^	−0.950 ± 0.105 ^b^	−0.733 ± 0.072 ^b^	−0.489 ± 0.062 ^b^	−0.605 ± 0.067 ^b^	−1.033 ± 0.087 ^b^	−0.722 ± 0.056 ^b^
Range (mm)	−0.3–1.0	−0.1–1.1	−0.3–1.7	−0.2–1.2	−0.1–0.8	−0.4–1.4	−0.6–1.8	−0.1–1.3
T3	−0.711 ± 0.061 ^c^	−0.717 ± 0.054 ^b^	−1.001 ± 0.101 ^b^	−0.772 ± 0.074 ^b^	−0.553 ± 0.053 ^b^	−0.667 ± 0.065 ^b^	−1.066 ± 0.081 ^b^	−0.789 ± 0.066 ^b^
Range (mm)	−0.3–1.1	−0.2–1.1	−0.3–1.7	−0.3–1.5	−0.1–0.8	−0.4–1.4	−0.6–1.8	−0.1–1.3
Statistical significance ^1^	*	*	*	*	*	*	*	*

^1^ based on ANOVA analyses, level of significance: * denotes *p* < 0.05; significant differences (*p* < 0.05) among groups (as demonstrated by post hoc tests) are indicated by different superscript letters (a, b, and c).

**Table 5:** Marginal bone level changes around individual implants in the mandible during the 42-month study period.

	Marginal Bone Level Changes (ΔBL) (mm ± SEM)							
Follow-Up	32DA (*n* = 18)	32MA (*n* = 18)	34DA (*n* = 18)	34MA (*n* = 18)	42DA (*n* = 18)	42MA (*n* = 18)	44DA (*n* = 18)	44MA (*n* = 18)
T1	−0.256 ± 0.051 ^a^	−0.550 ± 0.078 ^a^	−0.622 ± 0.052 ^a^	−0.494 ± 0.058 ^a^	−0.344 ± 0.054 ^a^	−0.422 ± 0.066 ^a^	−0.344 ± 0.054 ^a^	−0.538 ± 0.053 ^a^
Range (mm)	−0–0.6	−0.1–1.1	−0.2–1.0	−0.1–0.9	−0–0.8	−0.1–1.0	−0.2–1.0	−0.1–1.4
T2	−0.388 ± 0.053 ^b^	−0.689 ± 0.082 ^b^	−0.827 ± 0.053 ^b^	−0.678 ± 0.063 ^b^	−0.494 ± 0.046 ^b^	−0.627 ± 0.062 ^b^	−0.494 ± 0.046 ^b^	−0.722 ± 0.056 ^b^
Range (mm)	−0.1–0.7	−0.1–1.4	−0.4–1.3	−0.2–1.0	−0.1–0.8	−0.2–1.2	−0.3–1.1	−0.3–1.4
T3	−0.444 ± 0.051 ^c^	−0.722 ± 0.081 ^b^	−0.872 ± 0.044 ^b^	−0.717 ± 0.059 ^b^	−0.555 ± 0.051 ^b^	−0.694 ± 0.051 ^b^	−0.555 ± 0.051 ^b^	−0.789 ± 0.066 ^b^
Range (mm)	−0.1–0.8	−0.1–1.4	−0.6–1.3	−0.3–1.1	−0.2–0.9	−0.4–1.1	−0.3–1.3	−0.4–1.5
Statistical significance ^1^	*	*	*	*	*	*	*	*

^1^ based on ANOVA analyses, level of significance: * denotes *p* < 0.05; significant differences (*p* < 0.05) among groups (as demonstrated by post hoc tests) are indicated by different superscript letters (a, b, and c).

## Data Availability

All data generated during the study are presented in this paper.
